# Development of interactive algorithm for clinical management of acute events related to sickle cell disease in emergency department

**DOI:** 10.1186/1750-1172-9-91

**Published:** 2014-06-23

**Authors:** Gian Luca Forni, Gabriele Finco, Giovanna Graziadei, Manuela Balocco, Paolo Rigano, Silverio Perrotta, Oliviero Olivieri, Maria Domenica Cappellini, Lucia De Franceschi

**Affiliations:** 1 Department of Medicine, Section of Haematology, Ospedale Galliera, Genova, Italy; 2 Department of Medical Science “M. Aresu”, Section of Anesthesia, Intensive Care and Pain Therapy; University of Cagliari, Cagliari, Italy; 3 Department of Clinical Science and Community Health, University of Milan, Milan, Italy; 4 Fondazione IRCCS Ca' Granda Ospedale Maggiore Policlinico, Milano, Italy; 5 Department of Hematology and Oncology, Ospedali Riuniti Villa Sofia-Cervello, Palermo, Italy; 6 Department of Pediatrics, Second University of Naples, Napoli, Italy; 7 Department of Medicine, Section of Internal Medicine, University of Verona-AOUI-Verona, Verona, Italy

**Keywords:** Sickle cell disease, Acute vaso-occlusive crisis, Emergency departments, Pain

## Abstract

Sickle cell disease (SCD ORPHA232; OMIM 603903) is a rare hereditary red cell disorder, which global distribution is changed in the last decade due to immigration-fluxes from endemic areas to Western-countries. One of the main clinical manifestations of SCD are the acute painful vaso-occlusive crisis, which cause frequent accesses of SCD patients to the emergency departments (EDs). This has generated the requirement of feasible tools for emergency givers. In the context of the scientific-Italian-Society for the study of Thalassemias and Hemoglobinopathies (SITE), we developed an algorithm with interactive windows to guide physicians in managing SCD patients in EDs.

## Letters to the editor

**Figure 1:**
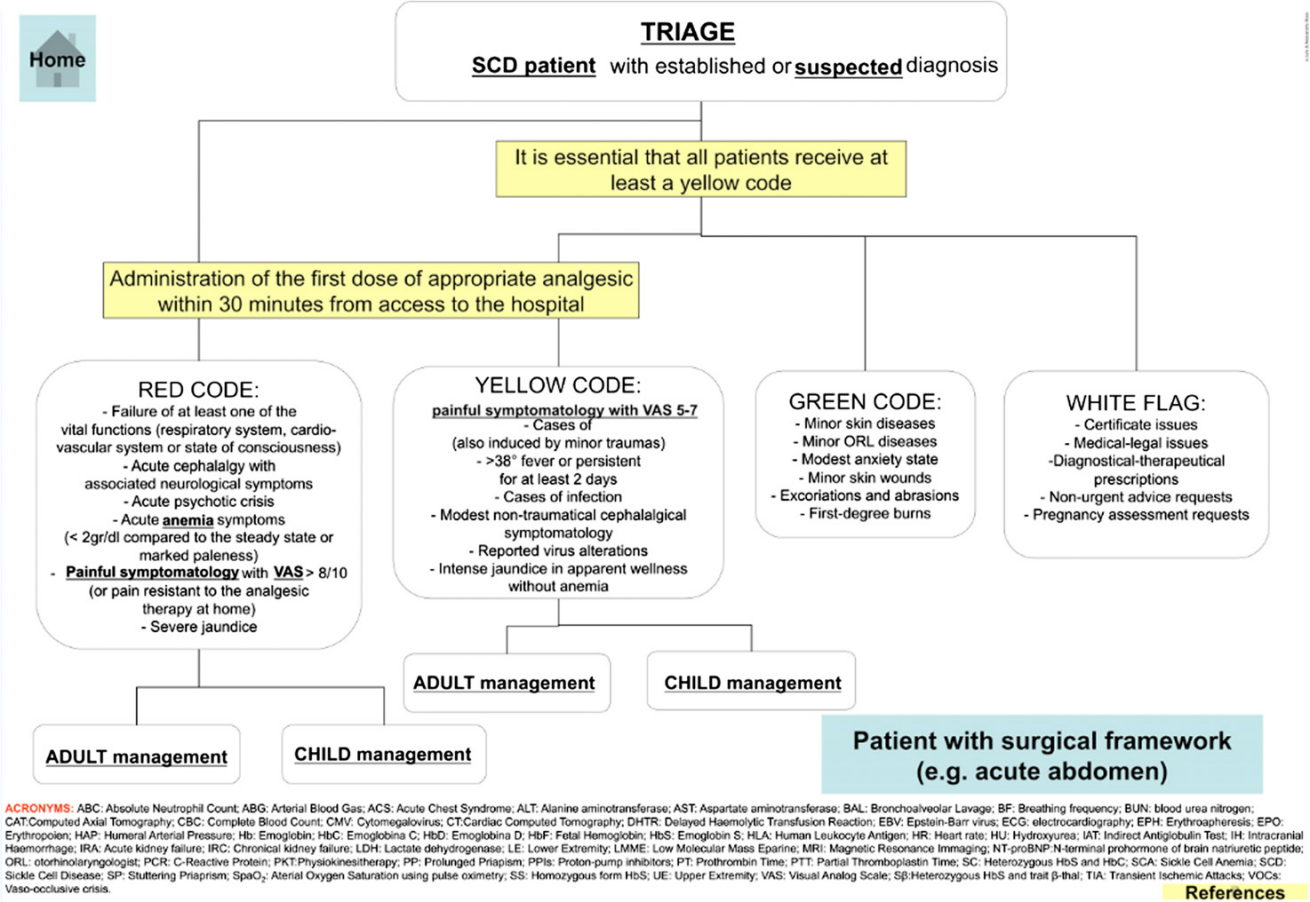
**The developed algorithm shows a series of windows on different clinical presentation of SCD patients at the EDs.** In the two blocked windows, we stressed the need to assign at least a triage code yellow to SCD patients in order to give an appropriate therapy for pain control within 30 minutes from patients arrival to the EDs. The boxes for triage each code link to pages, describing the details of the related therapy as shown in Figure 2 or the info about the differential diagnosis or the literature.

**Figure 2:**
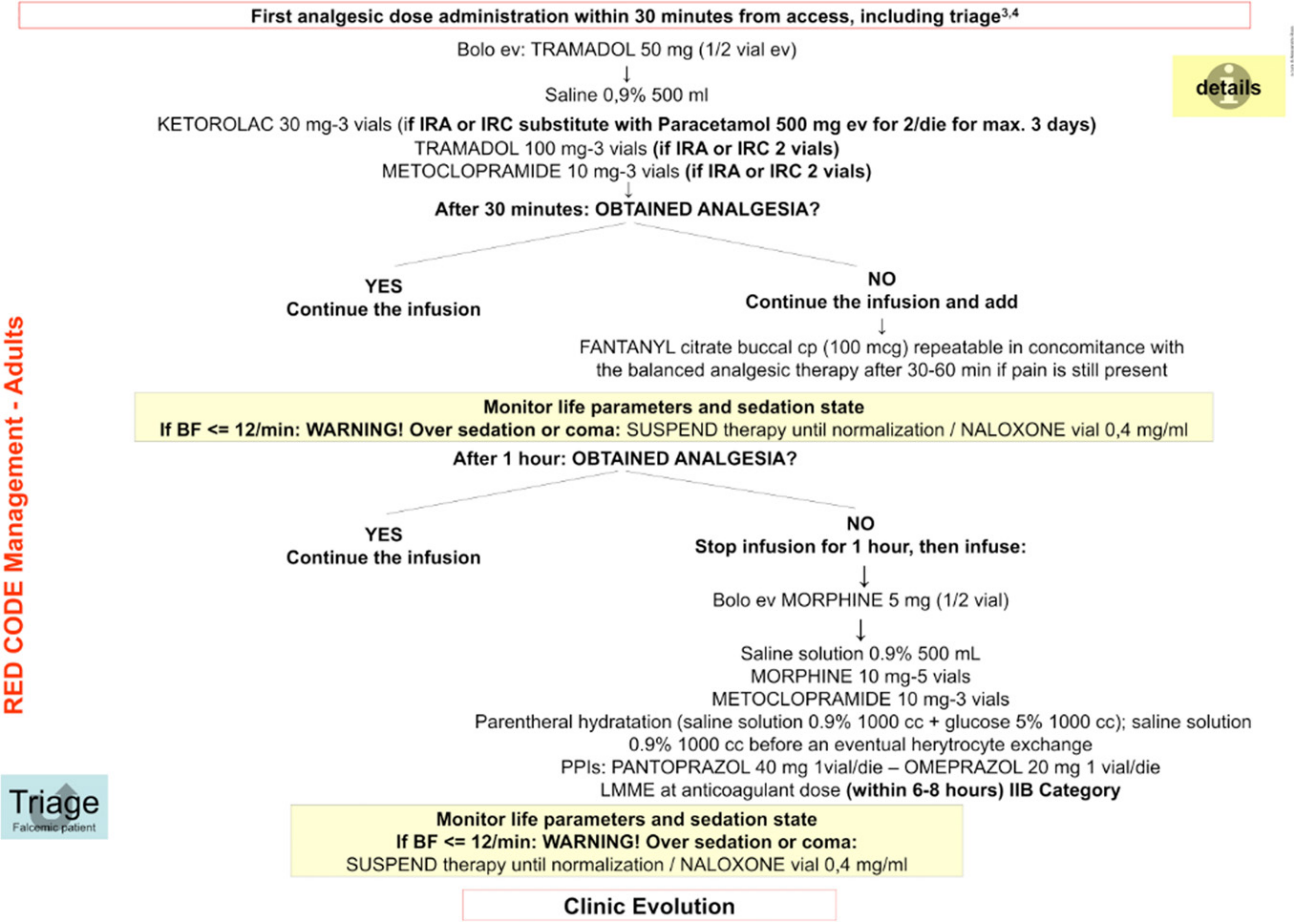
**This is an example of the working page opened by clicking on the “red code management” window.** The analgesic therapy is described in details and a flow-chart gives the timing and therapeutic choice. The page contains also one window named “details” containing a file with indications on the preparation and administration of the therapy. The button triage drives back to the home page.

Sickle cell disease (SCD ORPHA232; OMIM 603903) is a rare hereditary red cell disorder characterized by the presence of a point mutation on the β-globin chain, resulting in the synthesis of the pathological hemoglobin S (HbS) [1,2]. The two main clinical manifestations of SCD are chronic hemolytic anemia and acute painful vaso-occlusive crisis (VOCs), related to the entrapment of dense, dehydrated sickle red cells in the microcirculation [1,2]. Since in last two decades, the global distribution of SCD is largely changed due to immigration movements from endemic areas such as Africa to Western countries [3], the development of protocols for the management of acute events is crucially required by emergency givers [4,5]. In fact, SCD patients are the major users of emergency departments (EDs) compared to subjects with other severe hemoglobinopathies, generally feeling to be undertreated during VOCs [6,7]. Pain is the main symptom of VOCs and requires an intensive analgesic treatment. Previous studies have shown longer delay time to the initial analgesic treatment for SCD patients compared to subjects with other acute severe pain syndromes, despite the higher pain score at the admission to the EDs [8,9]. Ballas et al. have recently recommended an early and aggressive treatment of VOCs related to SCD similarly to the strategy developed for the management of acute ischemic stroke [5]. In addition, Glassberg et al. have pointed out that the management of acute VOCs related to SCD by emergency providers is more effective in presence of guidelines [9]. In this context, the scientific Italian Society for the study of Thalassemias and Hemoglobinopathies (SITE) has coordinated a national multidisciplinary working group that developed an algorithm to let the triage operators a fast and appropriate approach to the pediatrics and adult patients with SCD in EDs (Figure 1). The flow-chart of the algorithm has been designed to access, clicking on different buttons, to the detailed description of the diagnostic and therapeutic steps. Each one of the boxes of the algorithm from the green to the red code are interactive and linked to a detailed flow-charts similar to that shown in Figure 2. These flow-charts are evidences based designed and help in decision making processes and therapeutic management of acute VOCs related to SCD. The algorithm has also an introductive section on pathophysiology of SCD and extensive reference section, linked to PubMed in order to allow the physician to rapidly refer to the selected paper. The algorithm can be downloaded from the webpage of the SITE (http://www.site-italia.org) and can interface with the more diffuse hardwares.

Our algorithm has been developed to optimize the triage of SCD patients in order to ensure the right priority access to the evaluation and subsequent pain-control treatment within 30 min from patient arrival at the EDs. In fact, the time spent by the appearance of the first signs is an important prognostic index *quoad vitam* in SCD. SCD patients can access to the ED presenting full-blown manifestations acutely appeared, but often with a less painful symptoms, due to analgesic treatment at home [10,11]. In both cases, at the triage the patients should be early identified as SCD patients or as a patient with high degree suspicion for SCD related acute clinical complications. Thus, the algorithm helps in the correct identification of clinical pattern for SCD and assigns the yellow code (excluding cases with impairment of vital functions: red code) to SCD patients with acute painful crisis. The friendly interface of the developed algorithm also links to tables on (i) pain assessment scales for adults and children; (ii) management of acute chest pain or abdominal pain; (iii) management of SCD patients with acute neurological signs; (iv) peri-operative management of SCD patients with an acute surgical framework. The handiness and reliability of the algorithm was tested with the triage operators of two separate EDs and the final version was finalized using their feedback. The expected outcomes of this SITE action are to offer a fast and feasible tool to emergency providers and to improve the care of SCD during acute VOCs in the EDs where fast evaluation and management is required. The final goal is to prevent more acute clinical complications related to SCD that might worsen acute pain crisis, when they are not rapidly identified and treated [5].

## Abbreviations

HbS: Hemoglobin S; SCD: Sickle cell disease; VOCs: Vaso-occlusive crisis; EDs: Emergency departments.

## Competing interests

The authors have nothing to disclose.

## Author’s contributions

LDF, GLF and GF design the algorithm and wrote the text; MB, GG, PR developed the algorithm and contribute in writing the text; SP, MDC and OO contribute to the discussion and validation of the algorithm. All authors read and approved the final manuscript.
